# Three-dimensional graphene nanosheets as cathode catalysts in standard and supercapacitive microbial fuel cell

**DOI:** 10.1016/j.jpowsour.2017.03.135

**Published:** 2017-07-15

**Authors:** Carlo Santoro, Mounika Kodali, Sadia Kabir, Francesca Soavi, Alexey Serov, Plamen Atanassov

**Affiliations:** a Department of Chemical and Biological Engineering, Center Micro-Engineered Materials (CMEM), MSC01 1120 University of New Mexico, Albuquerque, NM, 87131, USA; b Department of Chemistry “Giacomo Ciamician”, Alma Mater Studiorum Universita’ di Bologna, Via Selmi 2, 40126, Bologna, Italy

**Keywords:** 3-D graphene nanosheets, Microbial fuel cell (MFC), Supercapacitive microbial fuel cell (SC-MFC), Power generation, Capacitance

## Abstract

Three-dimensional graphene nanosheets (3D-GNS) were used as cathode catalysts for microbial fuel cells (MFCs) operating in neutral conditions. 3D-GNS catalysts showed high performance towards oxygen electroreduction in neutral media with high current densities and low hydrogen peroxide generation compared to activated carbon (AC). 3D-GNS was incorporated into air-breathing cathodes based on AC with three different loadings (2, 6 and 10 mgcm^−2^). Performances in MFCs showed that 3D-GNS had the highest performances with power densities of 2.059 ± 0.003 Wm^-2^, 1.855 ± 0.007 Wm^-2^ and 1.503 ± 0.005 Wm^-2^ for loading of 10, 6 and 2 mgcm^−2^ respectively. Plain AC had the lowest performances (1.017 ± 0.009 Wm^-2^). The different cathodes were also investigated in supercapacitive MFCs (SC-MFCs). The addition of 3D-GNS decreased the ohmic losses by 14–25%. The decrease in ohmic losses allowed the SC-MFC with 3D-GNS (loading 10 mgcm^−2^) to have the maximum power (P_max_) of 5.746 ± 0.186 Wm^-2^. At 5 mA, the SC-MFC featured an “apparent” capacitive response that increased from 0.027 ± 0.007 F with AC to 0.213 ± 0.026 F with 3D-GNS (loading 2 mgcm^−2^) and further to 1.817 ± 0.040 F with 3D-GNS (loading 10 mgcm^−2^).

## Highlights

• Porous 3D Graphene nanosheets (3D-GNS) were fabricated using sacrificial templating.
• Activated Carbon (AC) and 3D-GNS investigated as cathode catalyst in microbial fuel cells.
• 3D-GNS showed higher activity towards oxygen electroreduction compared to AC.
• Performances in MFC with the 3D-GNS had a maximum of 2.059 ± 0.003 W m^−2^.
• SC-MFC with 3D-GNS at the cathode had a capacitance of 1.817 ± 0.040 F.

## Graphical abstract

**Figure:**
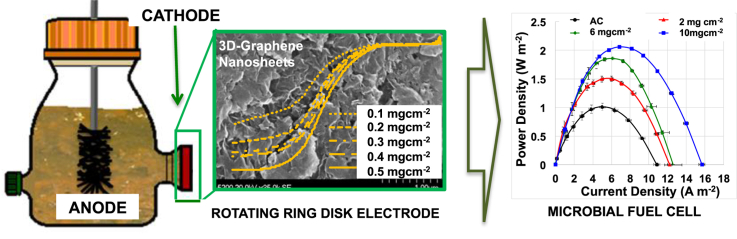

## 1 Introduction

Bioelectrochemical systems (BESs) are relatively novel devices that are designed for degrading organics [1], [2], [3] and/or producing value added products (VAPs) such as hydrogen [4], [5], [6], acetate [7], [8] or other interesting chemicals [9], [10], [11], [12] using lower energy than the traditional methods.

All BESs have an electroactive biofilm that is formed on the anode electrode that utilizes organics by oxidizing them and releasing the electrons and products of the oxidation reactions directly onto the solid support [13]. Microbial Fuel cell (MFC) belongs to the BESs category and it is probably the most investigated among BESs [13]. In MFCs, organics are oxidized at the anode while oxidants are reduced in the cathode compartment/electrode. A logical choice for oxidants is oxygen and this is dictated by the high potential of oxygen and the natural availability in atmosphere.

However, the reduction of oxygen has several limitations due to the unfriendly environment in which the reaction takes place. In fact, oxygen reduction reactions (ORR) follow a dual pathway depending on the catalyst and pH of the electrolyte. In acidic conditions, H^+^ is needed during the ORR. In alkaline media, OH^−^ is involved. During oxygen reduction reactions (ORR), water is reduced to H_2_O as final product in acid electrolyte, OH^−^ is formed in alkaline conditions. H_2_O and OH^−^ are formed if the reaction follows a 4e^−^ pathway when the oxygen is reduced completely [14], [15], [16]. When oxygen is only partially reduced, the electrochemical reaction follows a 2e^−^ pathway where H_2_O_2_ is formed [17], [18], [19]. However, the 4e^−^ pathway of oxygen electroreduction to H_2_O is preferred because double the no. electrons are accepted with half of the reactant (oxygen) utilized [20]. Also, H_2_O_2_ formation should be avoided because its high reactivity might negatively affect system stability. ORR taking place in neutral media in which both H^+^ and OH^−^ are in concentration of 10^−7^ M has not been studied in detail, and reaction mechanisms are therefore not fully understood.

The ORR kinetics in neutral media are further limited due to the low concentration of H^+^ and OH^−^ which causes high overpotentials and low kinetics [16]. In order to overcome these limitations, catalysts are often used to lower the overpotentials and accelerate the kinetics of the oxygen reduction reactions. By far, platinum has been the most used catalyst at the cathode of MFC working in neutral media [21], [22], [23]. The choice was dictated by the fact that Pt is often and successfully used at the cathode of traditional hydrogen/air polymeric electrolyte fuel cell (PEMFC) [24], [25] and direct methanol fuel cell (DMFC) [26], [27]. Unfortunately, working conditions are quite different not just for the MFCs operating in neutral pH electrolyte but also for the type of complex media in which MFCs operate. In fact, it has been established that anions bind with the catalysts deactivating Pt within few days of operation [28], [29], [30].

Hence, efforts have been directed towards the utilization of both amorphous as well as graphitized carbonaceous materials such as activated carbon (AC) [31], [32], [33], [34], [35], carbon nanofibers [36], [37], [38], modified carbon blacks [39], [40], graphene [41], [42], [43] and carbon nanotubes [44], [45], [46], [47] as cathode catalysts for ORR in microbial fuel cells. These graphitized nanostructures have a great potential to be utilized as ORR catalysts or as support materials due to their high surface areas, long term stability in polluted conditions, mechanical strength and more importantly, highly electrical conductivities [48], [49]. AC is the most utilized cathode catalyst in MFCs as showed by Wang et al. [22] due to a good compromise among low cost, high surface area and good electrical conductivity. Still, overpotentials remain high and the electrocatalytic activity in neutral media is questionable. Electrical conductivity can be even more enhanced by mixing carbon black with AC during the preparation of the cathode [50]. In literature, there are only a few number of studies that have utilized graphene materials at the cathode of MFCs [42], [43], [51]. Also, most graphitized materials fabricated using conventional techniques such as chemical vapor deposition or other electrochemical exfoliation techniques usually lack the porous morphology that is required for facilitating mass transport kinetics of oxygen and water into and out of the ORR active sites in the catalyst.

Another problem concerning MFCs is the quality of the current output that is not easily usable for practical applications. Despite several successful practical applications regarding MFCs are presented [52], [53], [54], MFCs are connected with external supercapacitors that allow to regulate the output and boost current/voltage to be used [55], [56], [57].

It has also been showed that intermittent operating mode allows to harvest a higher quantity of energy from working MFCs and a better utilization in practical applications [58], [59]. It has been shown previously the incorporation of internal supercapacitor inside MFCs in which the supercapacitive features of the MFC anode and cathode are exploited as negative and positive electrode of a supercapacitor [60], [61], [62], [63]. In those cases, MFC was working in pulsed and intermittent mode [60], [61], [62], [63]. The latter operating mode is preferable to deliver higher energy and power as previously demonstrated [58], [59]. Capacitive features of the bio-anode have also been reported in literature [64], [65], [66]. High surface area carbonaceous materials are deeply used as supercapacitive electrodes in bio-supercapacitors [67], [68], [69], [70], [71]. This is due to the fact that high surface area generates higher surface for the ion electrostatic attraction during the electrode charge. Several other metal oxides are used due to their high pseudocapacitive features [72]. Among them, ruthenium dioxide (RuO_2_) is the one with highest capacitance [73]. Ruthenium is not an earth abundant metal, is relatively expensive and consequently it cannot be used in large quantity in MFC systems in which the output is quite low and containing the costs is a priority.

In this work, three-dimensional graphene nanosheets (3D-GNS) were used as cathode catalysts for microbial fuel cells (MFCs) working in neutral operating conditions. The 3D-GNS catalyst was synthesized based on sacrificial support method (SSM) established previously [74], [75], [76], [77], [78]. In this method, amorphous silica was used as a sacrificial template incorporated into the graphene support matrix. The template was then subsequently etched out to produce the graphene nanosheets with a three-dimensional porous morphology (3D-GNS). The morphological and electrochemical properties of the 3D-GNS catalyst were studied using various surface analysis techniques. The catalytic activity was studied by rotating ring disk electrode (RRDE) technique. Analysis of peroxide production and the number of electron transferred allows to elucidate the mechanism of ORR. 3D-GNS was then incorporated into an air-breathing cathode mixed with AC and tested into a working MFC. At last, the supercapacitive feature of 3D-GNS incorporated into the air-breathing cathode was investigated into a supercapacitive MFC (SC-MFC).

## 2 Materials and method

### 2.1 3D-GNS preparation using the sacrificial support method (SSM)

Graphene oxide (GO) as a starting material was synthesized by the modified Hummers method [74]. The synthesized GO was fully exfoliated in a water solution using a high energy ultrasonic probe (700 kJ were delivered to 5 g of GO in 500 mL of de-ionized (DI) water for 1 h) followed by the addition of 10 g of fumed silica (Cab-O-Sil^®^, surface area ≈400 m^2^ g^−1^). The mixture of GO-SiO_2_ was ultrasonicated with the probe for additional hour and dried overnight at T = 85 °C on air. Using methods described previously [75], [76], [77], [78], the dry powder was ball-milled at 400 rpm for 15 min and subjected to reduction in 7% H_2_ (flow rate = 100 ccm) at T = 800 °C for 1 h. After reduction, this hybrid of GNS-SiO_2_ was ball-milled at 400 rpm for 15 min. The silica sacrificial support was leached by means of 40 wt % HF for 12 h, followed by continuous vacuum filtration until a neutral pH was achieved. The resulting 3D-GNS was dried overnight at T = 85 °C and was powdered and further pyrolyzed using N_2_ (flow rate = 100 ccm) at T = 850 °C for a duration of 2 h.

### 2.2 Surface chemistry and surface morphology

The morphologies of the synthesized materials were determined by scanning electron microscopy (SEM, Hitachi S-5200 Nano SEM with an accelerating voltage of 10 keV). The chemical composition of the 3D-GNS catalyst was further analyzing using Energy-dispersive X-ray spectroscopy (EDS).

### 2.3 Electrochemical characterization of 3D-GNS using rotating ring disk electrode technique

A rotating ring-disk electrode (AFE7R9GCPT, Pine Research. Co Ltd) with glassy carbon as the inner disk (Ø = 5.61 mm, A = 0.2472 cm^2^) and polycrystalline Pt (ID = 6.25 mm, OD = 7.92 mm, A = 0.1859 cm^2^) as the outer ring was used for the electrochemical testing of catalysts. The catalyst ink was prepared by adding 5 mg of the catalyst to 0.85 mL of a mixture of isopropanol and water with a ratio of 2:1 and 0.15 mL of 0.5 wt% Nafion. The inks were deposited onto the glassy carbon disk via drop casting method with a loading of 0.1, 0.2, 0.3, 0.4 and 0.5 mg cm^−2^. The disk electrode was used as the working electrode for the ORR in a neutral electrolyte solution of 0.1 M K-PB with 0.1 M KCl at pH 7.5. A graphite rod and Ag/AgCl (3 M KCl) electrodes were used as the counter and reference electrodes respectively. The electrolyte was purged and saturated with pure O_2_ for 45 min before starting the experiments. Linear sweep voltammograms (LSVs) were obtained by measuring the disk currents (I_disk_) while varying the potential from an initial value of +0.5 V vs. Ag/AgCl till −0.7 V vs. Ag/AgCl at 1600 rpm rotation speed with a scan rate of 5 mV s^−1^. The ring current (I_ring_) was measured for evaluating the peroxide yield (%H_2_O_2_) (eq. (1)) and the number of electron transferred according with equation (2) (eq. (2)).

(1) $$\%H_{2}O_{2}=\;\frac{200\times \frac{I_{ring}}{N}}{I_{disk}+\frac{I_{ring}}{N}}$$

(2) $$n=\frac{4I_{disk}}{I_{disk}+\frac{I_{ring}}{N}}$$

where I_disk_ is the disk current, I_ring_ is the ring current, n is the number of electrons transferred, and N is the collection efficiency (0.43).

### 2.4 Cathode preparation

Air breathing cathode configuration was used during this investigation. The preparation has been described in details in previous works [35]. In particular, activated carbon (AC, SX Ultra Sigma Aldrich, BET area equal to 845 m^2^ g^−1^), carbon black (CB) and polytetrafluoroethylene (PTFE) were grinded vigorously using a blender for at least 5 min. The percentage of each ingredient was 70 wt%, 10 wt% and 20 wt% respectively and it was chosen from previous optimization [50]. The grinded black powder was inserted into a metallic circular pellet die and pressed using a hydraulic press (Carver, USA) for 5 min at room temperature. Stainless steel mesh (MacMaster, USA) was used as current collector. AC-cathode was then obtained with a loading of 40 mg cm^−2^. Cathodes based on 3-D graphene nanosheets were prepared including 3D-GNS into the AC/CB/PTFE before pressing. The loadings selected of 3D-GNS were 2, 6 and 10 mg cm^−2^ respectively.

### 2.5 Cathode linear sweep voltammetry

The cathode was inserted into a lateral hole of modified glass bottle with the active part exposed to the solution and the SS mesh exposed to air. The bottle was filled with a solution of 0.1 M K-PB and 0.1 M KCl. The cathode was exposed to the solution overnight till the open circuit potential (OCP) was stable. Cathode polarization curve was run using linear sweep voltammetry (LSV) in a three-electrode configuration. In particular, LSV was run connecting the cathode to the working channel of the potentiostat, a titanium wire was immersed in the solution and used as counter. Ag/AgCl 3 M KCl was used as reference electrode. The scan rate used was 0.2 mV s^−1^.

### 2.6 MFC operation and electrochemical characterization

MFC operation was tested by using a mixture (50%–50%) of 0.1 M K-PB + 0.1 M KCl and activated sludge (Albuquerque Southeast Water Reclamation facility, Albuquerque, NM) as electrolyte. Sodium acetate (NaOAc) in concentration of 3 g L^−1^ was added into the solution as anodic food. Two cylindrical carbon brushes (3 cm diameter and 3 cm height each) were used as anode of the MFC. The anode was well operating and already colonized by electroactive bacteria. The anodes had been used for previous experiments [63]. High anode dimension was selected in order to make the cathode the limiting electrode in the system. The MFC was left in open circuit voltage (OCV) for at least 2 h till the OCV was stabilized. Polarization test was run from OCV to 0 mV staying each point for 5 min at constant predetermined potential. During the test, the cathode (used as working electrode) and the anode (used as counter electrode) potentials were monitored vs. the Ag/AgCl 3 M KCl reference electrode that was placed at equal distance by cathode and anode. The polarization test allowed constructing V-I and power curves, where power and current were normalized to the cathode area that was 2.85 cm^−2^.

### 2.7 SC-MFC operation and electrochemical characterization

The carbonaceous bio-anode and the oxygen air breathing cathode make the MFC a supercapacitive microbial fuel cell (SC-MFC). The anaerobic and aerobic environments at the bioanode and oxygen cathode, respectively, drive the electrode potentials towards negative (anode) and positive values (cathode). Electrochemical double layers (EDL) are formed at the interfaces of the polarized (charged) electrodes due to the presence of ions into the solution. The electrodes of the SC-MFC can be then rapidly and reversibly discharged by an electrostatic process. The subsequent cell rest permits to self-recharge the system.

Before the SC-MFC test, the cell was taken at rest for at least 24 h till the OCV was stable. The highest OCV featured by the SC-MFC is termed **V**_**max,OC**_. Galvanostatic (GLV) discharges were then performed at defined current pulses (**i**_**pulse**_) over a certain amount of time (**t**_**pulse**_). After the pulse, the SC-MFC was set in rest and, therefore, self-recharged to its initial voltage value (**V**_**max,OC**_). The GLV discharge caused an ohmic drop of the voltage (**ΔV**_**ohmic,cell**_) till the voltage **V**_**max**_ that is the maximum voltage that is featured by the SC-MFC during the discharge. **V**_**max**_ is here described by eq. (3):

(3) $$V_{max}=\;V_{max,OC}-\;\Delta V_{ohmic,\;cell}$$

**ΔV**_**ohmic,cell**_ is due to the ohmic losses of the system (anode, cathode and electrolyte). From the ohmic losses, it is possible to calculate the equivalent series resistance (ESR) of the cell by eq. (4):

(4) $$ESR=\;\frac{\Delta V_{ohmic,cell}}{i_{pulse}}$$

If anode and cathode profiles are measured distinctively and the reference electrode is placed at equal distance from anode and cathode, anode resistance (R_A_) and cathode resistance (R_C_) can be calculated according with Eqs. (5), (6):

(5) $$R_{A}=\;\frac{\Delta V_{ohmic,anode}}{i_{pulse}}$$

(6) $$R_{C}=\;\frac{\Delta V_{ohmic,cathode}}{i_{pulse}}$$

After the vertical drop, the voltage decreases with time like for a supercapacitor (**ΔV**_**cap,cell**_). The full discharge of the SC-MFC is reached when cell voltage reaches 0 V. The slope (**s** = dV/dt) of the voltage decrease over time can be taken to evaluate the apparent capacitance of the cell (**C**_**cell**_) following eq. (7):

(7) $$C_{cell}=\;\frac{i_{pulse}}{s}=\;\frac{i_{pulse}}{\frac{dV}{dt}}$$

Similarly, the slope of the cathode potential profiles (s_C_) during the time of discharge can be used to evaluate cathode capacitance (**C**_**C**_) by eq. (8):

(8) $$C_{C}=\;\frac{i_{pulse}}{S_{C}}=\;\frac{i_{pulse}}{\frac{dV_{cathode}}{dt}}$$

Power and energy are important indicators to define the performances of SC-MFCs. Maximum power (**P**_**max**_) was the higher value achievable at the beginning of the GLV pulse, i.e. without considering the **ΔV**_**cap,cell**_ decrease, and it can be calculated multiplying the current pulse (**i**_**pulse**_) and the maximum voltage (**V**_**max**_) according with eq. (9):

(9) $$P_{max}=\;V_{max}\;\times \;i_{pulse}$$

**P**_**max**_ is higher than the pulse power (**P**_**pulse**_) for a certain **t**_**pulse**_. **P**_**pulse**_ is calculated considering the energy delivered during the pulse (**E**_**pulse**_). The equation for obtaining **E**_**pulse**_ is here presented (eq. (10)):

(10) $$E_{pulse}=\;i_{pulse}\int_{0}^{t}Vdt$$

**P**_**pulse**_ is the ratio between **E**_**pulse**_ and **t**_**pulse**_ as shown in eq. (11):

(11) $$P_{pulse}=\;\frac{E_{pulse}}{t_{pulse}}$$

## 3 Results and discussion

### 3.1 Surface morphology/chemistry

**Fig. 1:**
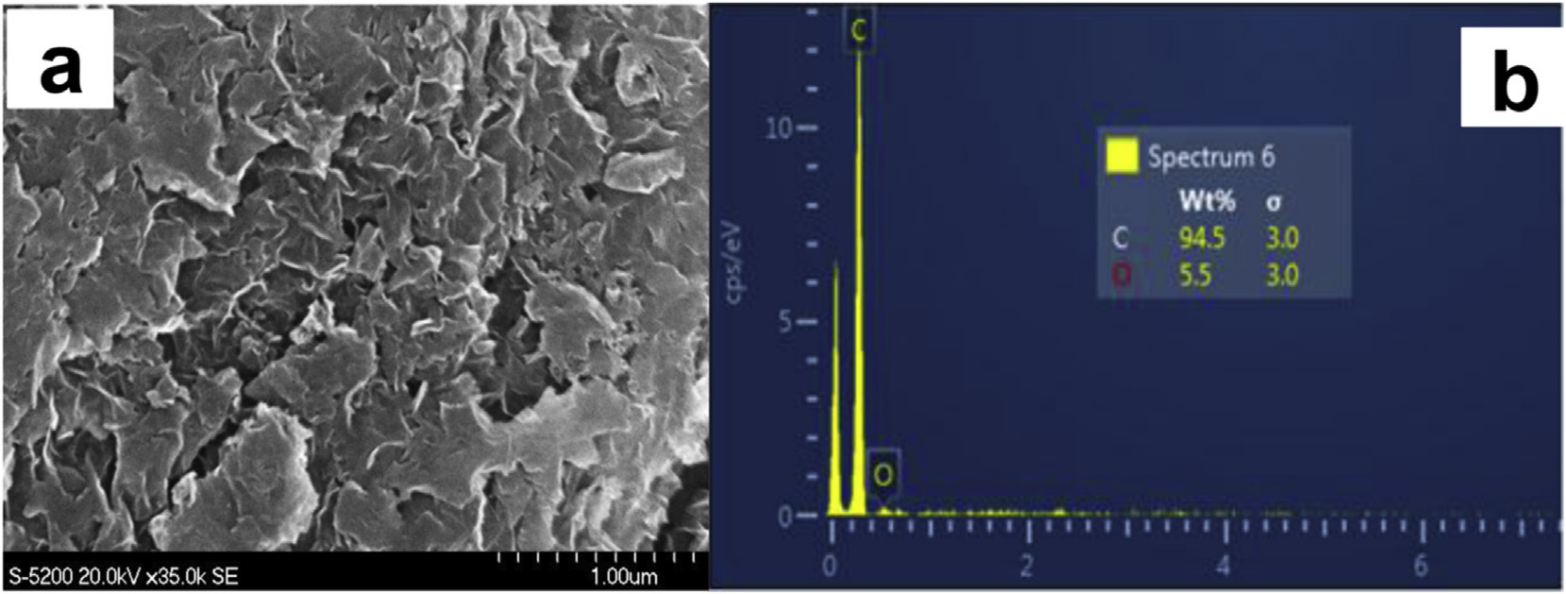
SEM micrograph of a three-dimensional graphene nanosheets (3D-GNS) (a) and energy-dispersive X-ray spectroscopy (EDS) of 3D-GNS (b).

As it can be seen from Fig. 1, the 3D graphene nanosheets (GNS), fabricated using the previously established sacrificial support method (SSM), which were etched into the matrix of graphene nanosheets during the leaching process described in Section 2.1), have a highly porous three-dimensional morphology. The BET surface areas of these highly crystalline 3D-GNS supports were previously shown to be ∼300–400 m^2^ g^−1^ [78].

The EDS analysis of the 3D-GNS supports (Fig. 1b) shows that only a small percentage of oxygen is present (∼5.5 at%) which could be due the presence of oxygenated functional groups such as carboxyls and quinone, etc. on the surface or edges of the graphene nanosheets.

### 3.2 RRDE measurements

**Fig. 2:**
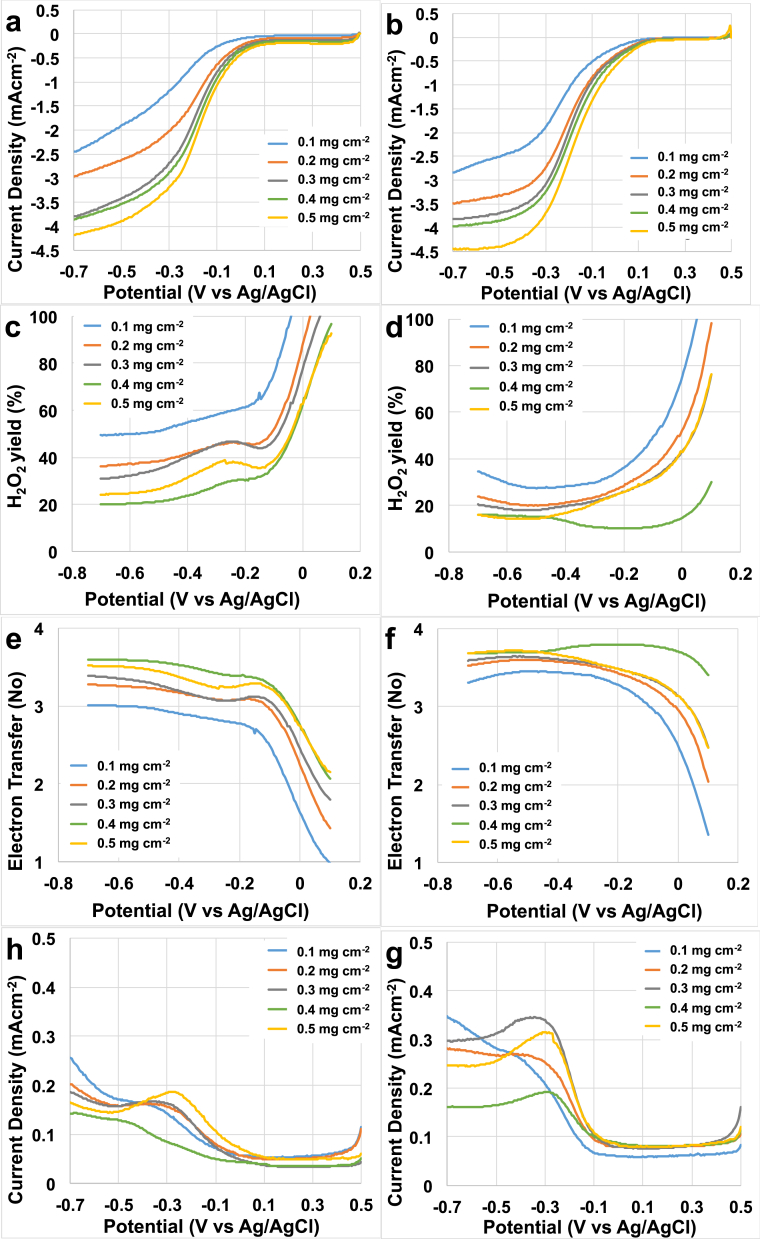
LSVs of AC (a) and 3D-GNS (b) in O_2_ saturated PBS 0.1 M at a rotation rate of 1600 rpm. % H_2_O_2_ produced by AC (c) and 3D-GNS (d) at different potentials. Number of electrons transferred in the ORR kinetics of AC (e) and 3D-GNS (f). Ring currents of AC (g) and 3D-GNS (h). Loadings of 0.1, 0.2, 0.3, 0.4 and 0.5 mg cm^−2^ were tested.

**Fig. 3:**
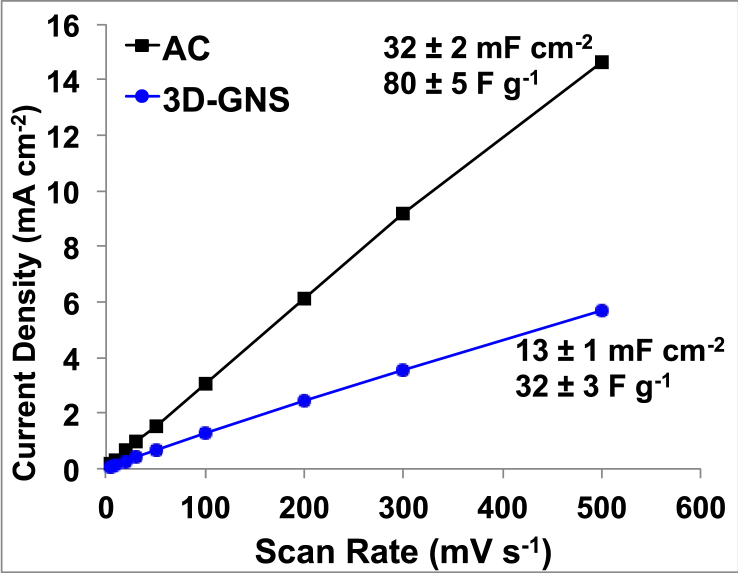
Relationship between scan rate and anodic current at 0.2 V vs. Ag/AgCl for 3D-GNS and AC (loading of 0.4 mg cm^−2^) at different scan rate and 0 rpm in O_2_ saturated K-PB 0.1 M solution.

LSV curves for AC (Fig. 2a) and 3D-GNS (Fig. 2b) were obtained at loadings of 0.1, 0.2, 0.3, 0.4 and 0.5 mg cm^−2^. All the LSVs are overlapped in Fig. S1. The parameters of interest in order to describe the catalytic performances of a material are: a) the electrocatalytic current onset potential; b) the half wave potential of the LSV; c) the limiting current. Half wave potentials are reported in Table S1. For loading of 0.1 mg cm^−2^, the onset potential was measured to be 0.13 V (vs Ag/AgCl) for 3D-GNS and −0.1 V (vs Ag/AgCl) for AC. The onset potential of 3D-GNS at 0.5 mg cm^−2^ (0.20 V) was also higher compared to AC (0.12 V), which indicates the facilitated ORR kinetics of 3D-GNS in comparison to AC at similar loadings.

The half wave potentials of 3D-GNS were also substantially higher compared to AC under similar loadings (Fig. S1). For example, at the highest catalyst loading (0.5 mg cm^−2^), the half-wave potential of 3D-GNS was estimated to be −0.16 V (vs Ag/AgCl) whereas AC had a lower half wave potential of −0.20 V. Similarly, the half wave potential of AC at the lowest catalyst loading (0.1 mg cm^−2^) was −0.3 V (vs Ag/AgCl) that was lower than 3D-GNS (−0.26 V vs Ag/AgCl) at the same loading conditions. Hence, it can be seen (Fig. S1), that an increase in catalyst loading led to an enhancement in the half wave potential and in the limiting current (Fig. 2a and 2b). Furthermore, under similar loadings, 3D-GNS consistently outperformed AC with higher onset as well as half wave potentials, which indicates its high catalytic performance towards ORR.

It is well known that ORR at carbonaceous metal-free materials is a 2e^−^ mechanism with production of peroxide as product of the reduction reaction [17]. Independently of the loading, peroxide yield was higher at high potentials, but decreased and stabilized at lower potentials (Fig. 2c and 2d). Generally, the peroxide production decreased with the catalysts loading increase from 0.1 to 0.4 mg cm^−2^ (Fig. 2c and 2d). A slight increase was detected when the loading increased from 0.4 to 0.5 mg cm^−2^ but still remained much lower than the peroxide produced at 0.1, 0.2 and 0.3 mg cm^−2^ (Fig. 2c and 2d).

Interestingly, at a catalyst loading of 0.1 mg cm^−2^ the peroxide production stabilized at ≈40–45% (Fig. 2c and 2d). The peroxide production decreased at 20–25% at higher loading of 0.4 and 0.5 mg cm^−2^ (Fig. 2c and 2d). Under similar loadings, 3D-GNS has the lowest hydrogen peroxide yield compared to AC, which could be due to its high degree of graphitization and its three-dimensional porous morphology.

Number of electrons transferred during the ORR was also calculated considering eq. (2) and showed in Fig. 2e and 2f. At lower loading (0.1 mg cm^−2^), the number of electron transferred was 3.0–3.2 that increased to 3.6–3.8 at catalyst loading of 0.4 and 0.5 mg cm^−2^ (Fig. 2e and 2f). The current registered at the ring is also shown (Fig. 2g and 2h). The reduction of peroxide produced with the increase of catalyst loading was probably due to a thicker layer of catalyst on the disk that traps the intermediate product of the reaction (H_2_O_2_). It can be speculated that the H_2_O_2_ is reduced to water inside the thicker catalyst layer on the disk. This speculation was demonstrated by the number of electron transferred during the ORR as showed in Fig. 2e and 2f that increased will 3.6–3.8 at higher loading. Consequently, those results gave us an important lesson on how to interpret the RRDE data in which the catalyst loading plays an important and key parameter. In fact, if the catalyst loading is high, peroxide production can be trapped within the layers and further reduced to water inside the disk without being able to be reduced and detected on the ring. If the catalyst layer is thick enough, a 2e^−^ mechanism can be confused as 4e^−^ mechanism leading the scientist to a mistake in the interpretation of the electron transfer mechanism.

The capacitive behavior of 3D-GNS was evaluated by cyclic voltammetry (CV) at different scan rates (5, 10, 20, 30, 50, 100, 200, 300 and 500 mV s^−1^). The CVs for AC and 3D-GNS at different scan rate are reported in Fig. S2. The cathodic peak is related to the oxygen reduction and for 3D-GNS, it took place always at higher potentials compared to AC (Fig. S2). The oxygen reduction peak is superimposed to the capacitive currents which are higher for AC than for 3D-GNS according to the higher specific surface area of AC respect to 3D-GNS. By dividing the capacitive current by the scan rate, it is possible to evaluate the electrode capacitances. Fig. 3 shows the linear trends of the anodic current at 0.2 V vs. Ag/AgCl with the scan rate which is representative of materials capacitive behavior. The slope of the plots gives areal capacitances of 32 ± 2 mF cm^−2^ and 13 ± 1 mF cm^−2^ for AC and 3D-GNS, respectively. These values correspond to 80 F g^−1^ and 32 F g^−1^ and suggest that the electrochemical accessible area of AC is more than double than that of 3D-GNS.

### 3.3 Cathode polarization curves in clean media

**Fig. 4:**
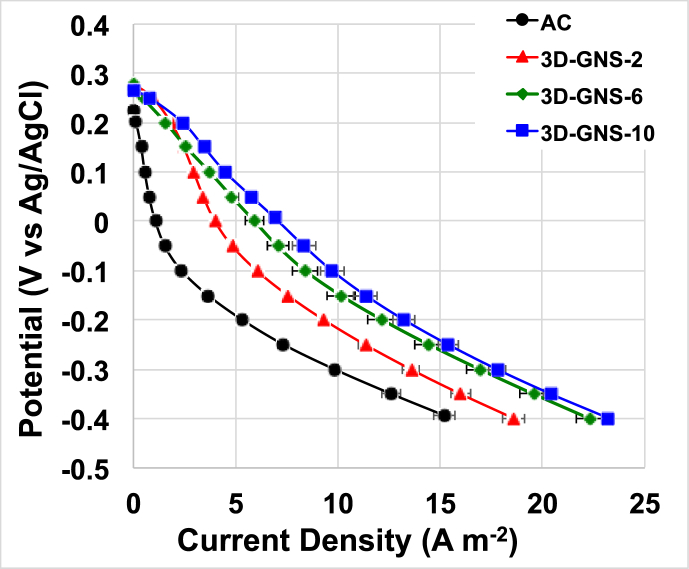
Cathode polarization curves run in clean neutral media for AC (black), 3D-GNS loading of 2 mg cm^−2^ (red), 3D-GNS loading of 6 mg cm^−2^ (green) and 3D-GNS loading of 10 mg cm^−2^ (blue) in MFC cell configuration. (For interpretation of the references to colour in this figure legend, the reader is referred to the web version of this article.)

The electrochemical performances of the new catalyst materials incorporated into air-breathing cathodes in MFCs were investigated using linear sweep voltammetry technique between OCP and −0.4 V vs. Ag/AgCl (Fig. 4). The results showed supremacy in electrocatalytic activity of 3D-GNS (loading 10 mg cm^−2^) over 3D-GNS (loading 2 and 6 mg cm^−2^) and simple AC (Fig. 4). This result is in agreement with the RRDE experiments (Fig. 2) that indicate higher performance of 3D-GNS compared with AC. Particularly, at −0.4 V vs. Ag/AgCl, 3D-GNS (loading 10 mg cm^−2^) produced a current density of 23.21 ± 0.03 A m^−2^ that was ≈4% higher than 3D-GNS (loading 6 mg cm^−2^), ≈15% higher than 3D-GNS (loading 2 mg cm^−2^) and ≈35% higher compared to AC. 3D-GNS (loading 6 mg cm^−2^) featured a current density measured of 22.3 ± 0.7 A m^−2^, 3D-GNS (loading 2 mg cm^−2^) featured a current density measured of 18.6 ± 0.6 A m^−2^ and AC has a current density measured of 15.2 ± 0.5 A m^−2^ respectively (Fig. 4).

### 3.4 Microbial fuel cells performances

**Fig. 5:**
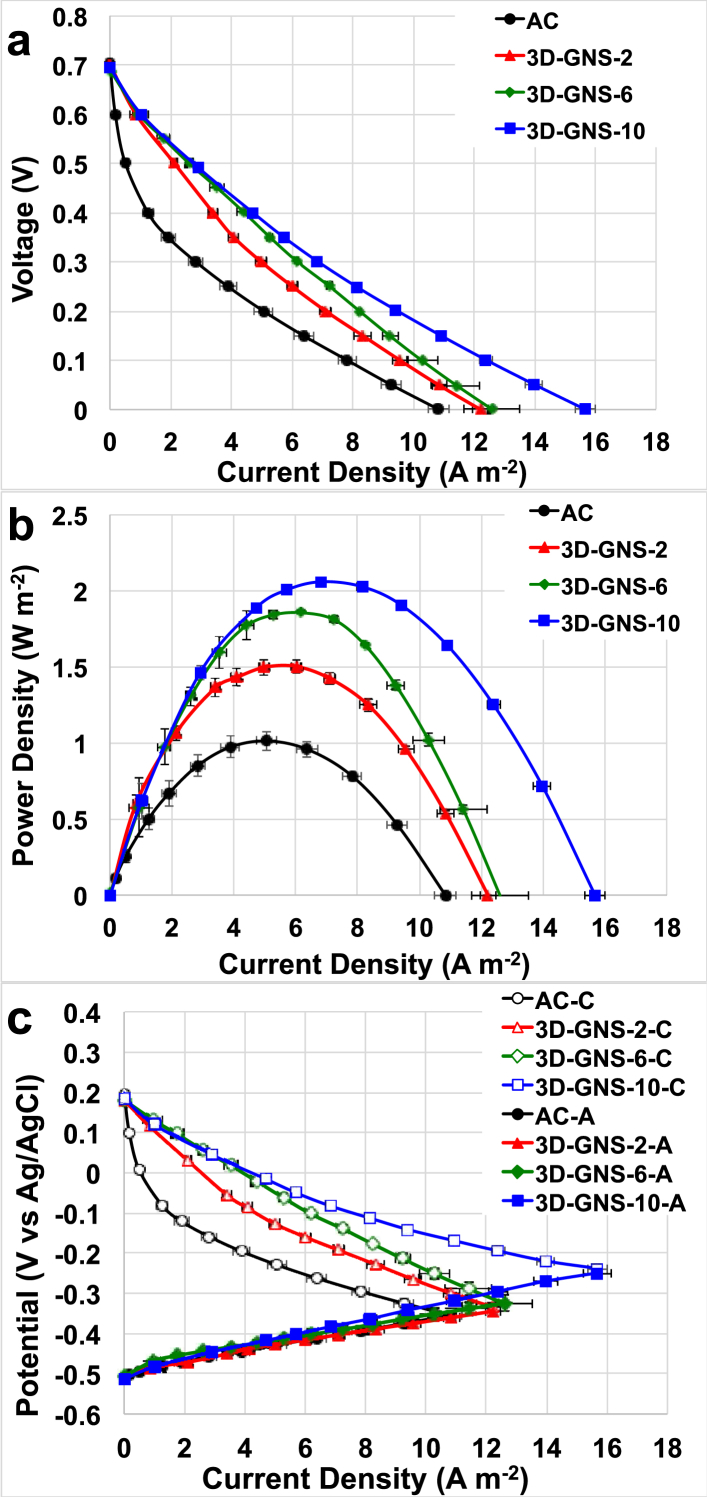
MFC cell Polarization curves (a), power curves (b) and cathode (-C) and anode (-A) (c) polarization curves for AC (black), 3D-GNS with a loading of 2 mg cm^−2^ (red, 3D-GNS-2), 3D-GNS with a loading of 6 mg cm^−2^ (green, 3D-GNS-6) and 3D-GNS with a loading of 10 mg cm^−2^ (blue, 3D-GNS-10). (C) (For interpretation of the references to colour in this figure legend, the reader is referred to the web version of this article.)

MFC performance followed strictly the trend identified in the previous sections related with RRDE analysis and LSV cathodes curves with 3D-GNS catalysts outperforming simple AC (Fig. 5). Similar open circuit voltage (OCV) was identified (Fig. 5a) that was due to: i) the contribution of the anode (OCP of −516 ± 6 mV vs. Ag/AgCl) that was similar for every MFC independently from the catalyst adopted and ii) the contribution of the cathode that was roughly +184 ± 10 mV vs. Ag/AgCl. Overall polarization curves indicated better performances for 3D-GNS catalyst (Fig. 5a) due to the better performing cathode polarization performance (Fig. 5c). The power generated for 3D-GNS cathode catalyst was 2.059 ± 0.003 W m^−2^, 1.855 ± 0.007 W m^−2^ and 1.503 ± 0.005 W m^−2^ for catalyst loading of 10, 6 and 2 mgcm^−2^ respectively (Fig. 5b). Peak of power of the cell with AC cathode catalyst was much lower and quantified in 1.017 ± 0.009 W m^−2^ (Fig. 5b). The catalyst loading affected the performance output and in this specific study the loading was increased from 2 mg cm^−2^ to 6 mg cm^−2^ and then to 10 mg cm^−2^. The MFCs featuring cathodes with 2, 6 and 10 mg cm^−2^ of 3D-GNS are labelled 3D-GNS-2, 3D-GNS-6, and 3D-GNS-10, respectively. The cell with AC is labelled AC.The performances increased by 19% when the 3D-GNS loading was triplicated and 29% when the 3D-GNS loading was quintupled.

### 3.5 SC-MFC complete discharges for i_pulse_ of 5 mA (17.5 A m^−2^)

The MFCs were studied as supercapacitive MFCs (SC-MFCs) in which the anode brushes and the air-breathing cathode were considered as the negative electrode and positive electrode of an internal supercapacitor, respectively. This was possible due to the self-polarization of the two electrodes due to the red-ox reaction of the system in which the acetate oxidation taking place at the anode bring the anodic potential towards negative values and the ORR brings the potential of the cathode to positive values. MFC operating in SC-MFC mode have been showed previously [60], [61], [62], [63].

**Fig. 6:**
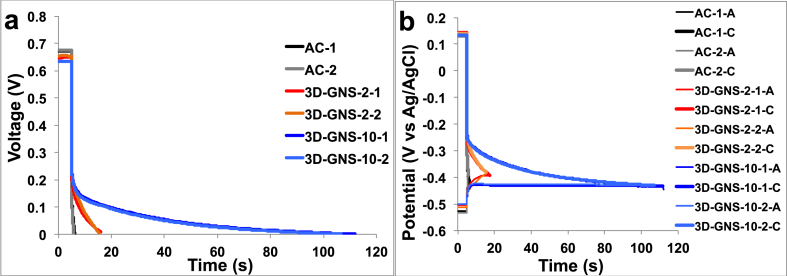
SC-MFC cell voltage (a) and electrode potential profiles (anode (A) and cathode (C) (b)) under full GLV discharges at i_pulse_ of 5 mA (17.5 A m^−2^) after 5 s of rest for AC (black), 3D-GNS loading of 2 mg cm^−2^ (red) and 3D-GNS loading of 10 mg cm^−2^ (blue). 1 and 2 indicates the number of trials. (For interpretation of the references to colour in this figure legend, the reader is referred to the web version of this article.)

Here, full GLV discharges at the high **i**_**pulse**_ of 5 mA (17.5 A m^−2^) are presented for the three different cathode catalysts, and cell voltage (Fig. 6a) and electrode potential profiles are shown (Fig. 6b). It can be noticed that during the discharge different trends can be detected in function of the catalysts utilized (Fig. 6). The main feature is that discharges last more when 3D-GNS is used and the effect is more pronounced at the higher loadings. The discharge times are reported in Table 1 which also reports cell and electrode capacitances and resistances evaluated by the GLV test at 5 mA (17.5 A m^−2^). The longer duration of the discharge is mainly related to a slower decrease of cell voltage over time, hence to what we can define a higher “apparent” capacitance (C_cell_) of the SC-MFC with 3D-GNF with respect to that with AC (see eq. (7) and eq. (8)). The C_cell_ was measured in 1.817 ± 0.040 F for 3D-GNS-10, 0.213 ± 0.026 F for 3D-GNS-2 and 0.027 ± 0.007 F for AC. C_C_ varied significantly with the cathode utilized and affected the overall capacitance. Particularly, 3D-GNS-10 had a C_C_ of 1.955 ± 0.035 F that was 5.7 times higher than 3D-GNS-2 (0.351 ± 0.020 F) and 45 times higher than AC (0.043 ± 0.002 F) (Table 1). Also in this case interestingly, the loading of the 3D-GNS affected positively the cathodic capacitance (Table 1). The addition of 3D-GNS increased significantly the cathode capacitance benefiting also the overall cell capacitance and the performances output. All these data cannot be explained referring to only-capacitive (electrostatic) discharges occurring at the cathodes. Indeed, as shown in Fig. 3 and discussed in Section 3.2, the specific capacitance of 3D-GNS is lower than the one of the AC. The addition of 2 mg cm^−2^ or 10 mg cm^−2^ of 3D-GNS to 40 mg cm^−2^ of AC cannot justify the 5.7 and 45 higher capacitive response of 3D-GNS based cells with respect to that with AC. Differently, a hybrid discharge that includes faradic and capacitive processes can be claimed. The high catalytic activity of 3D-GNS enables oxygen reduction even at such high currents as 5 mA. This is the faradic process that is superimposed to the capacitive discharge of the polarized carbon surface and that enables long discharges and high apparent C_cell_.

**Table 1:** Discharge times (t_discharge_), cell (C_cell_) and cathode (C_C_) capacitance, cell equivalent series resistance (ESR) and anode (R_A_) and cathode (R_C_) resistances evaluated by full discharges at i_pulse_ 5 mA (17.5 A m^−2^) of the SC-MFCs.

	trial	t_discharge_ s	ESR Ω	R_A_ Ω	R_C_ Ω	C_cell_ F	C_C_ F
AC	1	1.2	96.8	12.5	83.4	0.032	0.042
AC	2	0.9	93.4	6.7	84.8	0.022	0.045
3D-GNS-2	1	12.4	76.0	2.0	74.0	0.231	0.365
3D-GNS-2	2	10.5	76.9	3.8	73.0	0.194	0.337
3D-GNS-10	1	107.0	68.7	2.6	64.1	1.845	1.955
3D-GNS-10	2	101.9	69.8	3.5	65.3	1.788	1.930

A careful analysis of the curves at the shorter times indicates that ESR lowers with the addition 3D-GNS which also contributes to increase discharge times and this is probably due to the increase in electrical conductivity of the material (Table 1). In fact, the cell with 10 mg cm^−2^ of 3D-GNS has an ESR of 69.3 ± 0.8 Ω followed by that with 2 mg cm^−2^ of 3D-GNS with 76.5 ± 0.6 Ω and that with AC featuring 95 ± 2 Ω (Fig. 6 a and Table 1). R_C_ was different and depended on the cathode catalyst utilized (Fig. 6 b and Table 1) while R_A_ was roughly similar varying from 2 to 12.5 Ω (Table 1). R_C_ was the highest contribution to the overall ohmic resistance. Particularly, 3D-GNS-10 had an R_C_ of 64.7 ± 0.8 Ω followed by 3D-GNS-2 with R_C_ of 73.5 ± 0.7 Ω and AC (85 ± 2 Ω) (Fig. 6b). This indicates that the addition of 3D-GNS decreased the ohmic resistance of the cathodes giving a benefit of the overall performances. Also in this situation, the increase in loading leads to a slight decrease in the cathode ohmic resistance (Table 1). However, even with 3D-GNS the cell ohmic drop is very high and is the main parameter that limits discharge duration at high currents.

### 3.6 SC-MFC power curves

**Fig. 7:**
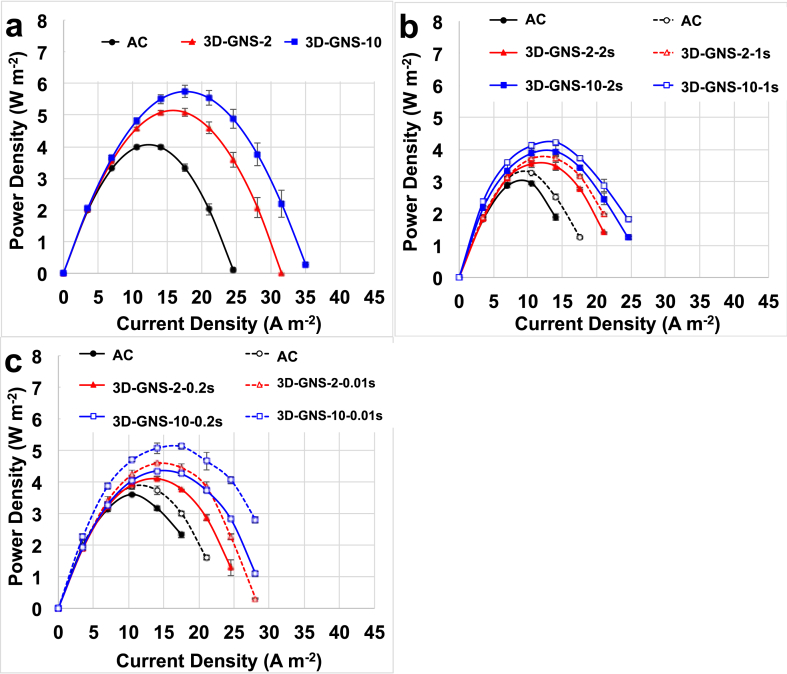
**P**_**max**_ (a) and **P**_**pulse**_ for **t**_**pulse**_ of 2 s and 1 s (b) and 0.2 s and 0.01 s (c). Colors are black for AC, red for 3D-GNS with loading of 2 mg cm^−2^ and blue for 3D-GNS with loading of 10 mg cm^−2^. (For interpretation of the references to colour in this figure legend, the reader is referred to the web version of this article.)

**P**_**max**_ and **P**_**pulse**_ for **t**_**pulse**_ of 2 s, 1 s, 0.2 s and 0.01 s for the three materials investigated are given in Fig. 7. **P**_**max**_ was the highest value obtained since it is calculated without considering the capacitive decrease of the SC-MFCs voltage (Fig. 6a). **P**_**max**_ for SC-MFC with 3D-GNS-10 was 5.746 ± 0.186 W m^−2^ that was 13% higher than that of the cell with 3D-GNS-2 (5.088 ± 0.124 W m^−2^) and 32% higher than that of the cell with AC cathode (3.986 ± 0.079 W m^−2^) (Fig. 7a). Higher **P**_**pulse**_ were achieved with lower **t**_**pulse**_ (Fig. 7b and 7c). Maximum **P**_**pulse**_ for **t**_**pulse**_ of 10 ms was 5.121 ± 0.077 W m^−2^ for 3D-GNS-10, 4.589 ± 0.020 W m^−2^ for 3D-GNS-2 and 3.865 ± 0.093 W m^−2^ for AC. 3D-GNS-10 had a maximum **P**_**pulse**_ of 4.325 ± 0.037 W m^−2^, 4.225 ± 0.039 W m^−2^, 3.937 ± 0.169 W m^−2^ for **t**_**pulse**_ of 0.2 s, 1 s and 2 s respectively. 3D-GNS-2 had lower maximum **P**_**pulse**_ quantified in 4.105 ± 0.049 W m^−2^, 3.712 ± 0.109 W m^−2^, 3.547 ± 0.112 W m^−2^ for **t**_**pulse**_ of 0.2 s, 1 s and 2 s respectively. Lowest maximum **P**_**pulse**_ was achieved by AC and it was 3.608 ± 0.033 W m^−2^, 3.279 ± 0.022 W m^−2^, 2.942 ± 0.052 W m^−2^ for **t**_**pulse**_ of 0.2 s, 1 s and 2 s, respectively. Also in this case, the supremacy in output for SC-MFC with 3D-GNS cathode catalyst with the highest loading (10 mg cm^−2^) was shown. Performances were then followed by SC-MFCs with 3D-GNS cathode catalyst with loading of 2 mg cm^−2^ and SC-MFCs with AC.

## 4 Outlook

PGM-free catalysts based on earth abundant metals or carbonaceous metal-free materials are necessary as Pt substitute for ORR in neutral media. In this work, we present the performances of 3D graphene nanosheets (3D-GNS) as metal-free catalysts for cathode MFCs. RRDE analysis showed that a thick layer on the disk might hinder the real 2e^−^ transfer mechanism happening during the ORR. Consequently, a thin layer is suggested in order to avoid misinterpretations. In any loading investigated, 3D-GNS outperformed AC. Electrochemical results of the catalyst incorporated in air-breathing cathode electrode indicated that the addition of 2, 6 and 10 mg cm^−2^ of 3D-GNS to the AC air-breathing cathode improves significantly the power output by 50%, 85% and 100%, respectively. Interestingly, the higher power densities achieved in this investigation using carbonaceous metal-free materials as catalysts were similar to the power densities obtained in previous works using Fe-N-C materials under the same operating conditions [79], [80], [81], [82]. This means that in these operating conditions, 3D-GNS can be an alternative to PGM-free catalysts incorporated into air breathing cathodes. Further investigations should address the effect of 3D-GNS and PGM-free catalysts merged together for ORR. The use of 3D-GNS catalyst led to a light increase in the overall cathode capital cost but the advantage obtained in the performances was quite evident and further scale up should consider additional decrease in manufacturing cost for large-scale 3D-GNS production. Existing literature showed that discontinuous MFC operation is actually more efficient than continuous mode [58], [59] indicating that the utilization of the MFC electrodes as the electrodes of an internal supercapacitor is a promising technique for harvesting successfully energy. The addition of 3D-GNS into the cathode increased the hybrid capacitive feature of the positive electrode of the supercapacitive-MFC. 3D-GNS decreased significantly the ohmic resistance of the cathode lowering the ESR and enhanced the capacitance of the SC-MFC. However, improvements in ESR are still needed in order to get high cell voltage operation under high current pulses. An “apparent” capacitance of 1.817 ± 0.040 F was measured in the case of 3D-GNS with a loading of 10 mg cm^−2^. This is the first time ever that such high capacitance with order of magnitudes of Farads is shown for a microbial fuel cell used as a supercapacitor with aqueous electrolyte. This indicates the possibility of utilizing microbial fuel cell to deliver energy/power pulses with high quality current of interest for practical applications. In agreement with previously reported data, current/power pulses from a SC-MFC were much higher compared to the current/power obtained from MFCs. Supercapacitive pulse mode compared to continuous operation and external supercapacitor integration has the advantage of enabling a a simpler and more compact system.

## 5 Conclusions

Three-dimensional graphene nanosheets (3D-GNS) were used as novel catalysts for air-breathing cathode MFCs based on AC cathode. 3D-GNS catalyst was compared with AC used as control. 3D-GNS increased the electrocatalytic activity of the cathode during ORR. Additionally, it was shown that the catalysts loading increase from 2 mg cm^−2^ to 6 mg cm^−2^ and then 10 mg cm^−2^ positively affects the performance. The highest power output was measured for 3D-GNS-10 and it was 2.059 ± 0.003 W m^−2^ followed by 3D-GNS-6 with 1.855 ± 0.007 W m^−2^, then 3D-GNS-2 with 1.503 ± 0.005 W m^−2^ and AC (1.017 ± 0.009 W m^−2^). 3D-GNS was also tested as supercapacitive material in SC-MFC. 3D-GNS used at the cathode decreased significantly the ohmic resistance and enhanced the cathode and the cell capacitance during SC-MFC discharge. Also in this case, higher loading played a positive role. An “apparent” capacitance of 1.817 ± 0.040 F was achieved when 3D-GNS with loading of 10 mg cm^−2^ was used. Consequently, maximum power for SC-MFCs was 5.746 ± 0.186 W m^−2^ with 3D-GNS-10, 5.088 ± 0.124 W m^−2^ with 3D-GNS-2 and 3.986 ± 0.079 W m^−2^ with AC.
