# Supplementary material for: Three-dimensional graphene nanosheets as cathode catalysts in standard and supercapacitive microbial fuel cell
Source: J Power Sources. 2017 Jul 15;356:371–80. doi: 10.1016/j.jpowsour.2017.03.135 (PMC5465940; doi:10.1016/j.jpowsour.2017.03.135)
Supplement: Supplementary file 1 [file mmc1.docx]

**Supporting Information**

**Three-Dimensional Graphene Nanosheets as Cathode Catalysts in Standard and Supercapacitive Microbial Fuel Cell**

Carlo Santoro^1^, Mounika Kodali^1^, Sadia Kabir^1^, Francesca Soavi^2^, Alexey Serov^1^, *Plamen Atanassov^1^

^1^ Department of Chemical and Biological Engineering, Center Micro-Engineered Materials (CMEM), MSC01 1120 University of New Mexico Albuquerque, New Mexico 87131, USA

^2^ Department of Chemistry “Giacomo Ciamician”, Alma Mater Studiorum Universita’ di Bologna, Via Selmi 2, 40126, Bologna, Italy

*Corresponding author: [plamen@unm.edu](mailto:plamen@unm.edu)

Center for Micro-Engineered Materials (CMEM), Department of Chemical & Biological Engineering, University of New Mexico, Albuquerque, NM 87131, USA.

**Figure S1**. Disk current produced by AC (black) and 3D-GNS (red) in O_2_ saturated PBS 0.1 M at a rotation rate of 1600 rpm. Loadings of 0.1, 0.2, 0.3, 0.4 and 0.5 mg cm-2 were tested.

**Table 1.** Half wave potential for AC and 3D-GNS catalyst at loading of 0.1, 0.2, 0.3, 0.4 and 0.5 mg cm^-2^.

| **catalyst** | **E _1/2_** | |
| --- | --- | --- |
| **loading** | **AC** | **3D-GNS** |
| **(mg cm^-2^)** | **V (vs Ag/AgCl)** | |
| **0.1** | -0.30 | -0.26 |
| **0.2** | -0.35 | -0.22 |
| **0.3** | -0.22 | -0.20 |
| **0.4** | -0.21 | -0.19 |
| **0.5** | -0.20 | -0.16 |

**Figure S2.** CVs from 0.5 V (vs Ag/AgCl) to -0.7 V (vs Ag/AgCl) at different scan rate for AC (a and b) and 3D-GNS (c and d).
